# The Thermal Parameters of Mortars Based on Different Cement Type and W/C Ratios

**DOI:** 10.3390/ma13194258

**Published:** 2020-09-24

**Authors:** Agata Stolarska, Jarosław Strzałkowski

**Affiliations:** Department of Building Physics and Building Materials, Faculty of Civil Engineering and Architecture, West Pomeranian University of Technology Szczecin, Piastów 50, 70-311 Szczecin, Poland; jstrzalkowski@zut.edu.pl

**Keywords:** cement mortar, thermal conductivity, volume specific heat, total porosity, total surface area, MIP, water absorbability

## Abstract

This study examines the thermal parameters of mortars based on different cement type and water-cement W/C ratios. The presented relationships are important from the point of view of thermal insulation of the entire building component, of which the mortar is a part. The thermal properties of the mortar, and in particular its dependence on the degree of moisture, is important information from the point of view of hygrothermal simulations of building components. The moisture effect on the thermal properties was tested using nine mortar types. The study consisted of producing nine types of mortar on the basis of three cements (CEM I 42.5R, CEM II A-S 52.5N, CEM III A 42.5N). For each cement type, three variants of specimens were prepared which differed according to their water/cement ratio (0.50, 0.55 and 0.60). The main research of thermal parameters was carried out using a non-stationary method based on the analysis of changing heat flux readings. The thermal conductivity, volume-specific heat and thermal diffusivity values were analyzed. The tests performed allowed for determination of the density of specimens, water absorbability and thermal parameters in three water saturation states: dry, natural and wet. Additional microstructural tests were performed using mercury intrusion porosimetry. The obtained parameters were used to determine the relationship between the measured properties. An adverse effect of dampness on the thermal insulation of the studied materials was confirmed. In extreme cases, the increase in thermal conductivity due to material high moisture was 93%. The cement used affects the relationship between the total specific surface area and the W/C ratio. As expected, the total porosity of specimens was higher for mortars with higher W/C ratios. A strong correlation has been demonstrated between the total surface area and thermal conductivity. The opposite results were obtained when assessing the relationship between the total specific surface area and water absorbability. In case of specimens CEM II A-S 52.5N, the relation was the proportional, and in specimens CEM III A 42.5N, the relationship was inversely proportional to the W/C ratio.

## 1. Introduction

One of the most important economic challenges in recent years is the reduction of energy consumption. This has resulted in the need for improved methods of precise hygrothermal calculations. Thermal conductivity coefficient is the key parameter based on which building materials are selected in terms of thermal insulation. The aim of achieving its lowest possible value is to produce materials with better thermal parameters in order to provide users with maximum comfort while minimizing heating costs. Cement mortar, as a component of building partitions, must also meet those requirements. Mechanical and physical properties of cement mortars have been the subject of intensive research in recent years [1,2,3,4,5]. The techniques of measuring microstructure parameters and thermal conductivity are discussed. Also, the structure of these materials is characterized and various models for the description of thermal conductivity are discussed. The results of research and the effects of admixtures, different densities, porosities and microstructures on thermal conductivity are also presented. Moisture, the type of admixture used, the amount of aggregate and the age of the concrete have a significant influence on the thermal conductivity of concrete or mortar.

However, moisture in the material plays a key role. In the case of concrete, the thermal conductivity increases with increasing moisture content and, according to Reference [6], it is 70% higher when wet than when dry. In the mentioned article [6], the research on thermal conductivity of mortar, concrete and its main component aggregate is presented. The thermal conductivity of mortar and concrete was measured at different moisture contents. The relation between the conductivity of concrete and the conductivity of aggregate at various saturation levels, including dry and fully saturated ones, has been worked out. It was confirmed that the thermal conductivity of aggregate and mortar increases with increasing humidity and the type of aggregate has a significant impact on the thermal conductivity of mortar and concrete. In the case of mortar, a decrease by 38% in thermal resistance between the dry and wet state of specimens was observed [6]. The study of light mortar with expanded perlite showed 60–80% reduction of thermal conductivity at 75–100% substitution of fine aggregates [7].

Zhang et al. [8] proved that the thermal conductivity coefficient of cement mortar and concrete in its saturated state is about 70% and 50% higher respectively, than in its dry state. For example, in Reference [9], mortars with three different water to cement ratio (W/C) indices of 0.4, 0.5 and 0.6 were tested. The researchers showed that the value of thermal conductivity of cement mortar made from volatile ash with light aggregate for dry and saturated specimens was 0.16 and 0.31 W/(mK). Seven factors influencing the thermal conductivity of concrete, mortar and cement paste were evaluated in Reference [10]. They were age, water to cement ratio (W/C), types of admixtures, volume fraction of aggregate, fraction of fine aggregate, temperature and humidity. The aggregate volume fraction and moisture content of the tested specimens proved to be the main factors influencing the thermal conductivity of concrete. The conductivity of mortar and cement paste was strongly influenced by the ratio of W/C and the type of admixture.

In Reference [11], in addition to the influence of the total volume of pores and the distribution of pores, the influence of moisture content on thermal conductivity was also evaluated. Thermal conductivity of cement-based composite material was measured using the impulse technique, ranging from dry to fully saturated with water. The accuracy of the techniques used was evaluated by comparing the measured and calculated results. Measurements of the thermal conductivity coefficient were also the subject of this study [8]. Among others, cement mortar with a ratio of W/C = 0.50 was tested. The guarded hot plate method has been selected for the measurement of thermal conductivity. Extensive research on mortars was carried out in Reference [12]. The influence of moisture content on thermal conductivity of 17 different thermal mortars was evaluated. Furthermore, on the basis of standards and experimental measurements, the correlation between thermal conductivity was evaluated. Aside from studies constituting a review of thermal conductivity measurement techniques [13,14], the assessment of the possible application of existing models to predict thermal conductivity of cement-based composites is also conducted. For example, in Reference [15], the selected models describing the heat conductivity coefficient in porous, dry and damp building materials were analyzed. The values obtained from the models were compared with the measured parameters. 

Mechanical and physical parameters of mortars with various additives are also tested. For example, Reference [16,17] concerned the partial replacement of Portland cement with rice husk ash, and with the addition of industrial waste [18,19]. Gomes et al. researched the thermal conductivity of thermal insulating mortars with expanded polystyrene EPS and silica-based aerogel [20]. The mortars were evaluated in dry state and at different moisture content levels. Moreover, several methods were performed: two steady-state and two transient methods. The research used, among other methods, the Applied Precision ISOMET 2114 apparatus, which was also used in this study.

Sakir et al. [21] prepared a comprehensive review of the use of supplementary cementitious materials in cement mortars. Based on the results, the water to cement ratio affects the porosity and strength of the mortar. The lower W/C ratio provides greater strength and reduced porosity. At a constant W/C ratio, the fragmentation of the binding materials affects the porosity and pore size distribution in cement-based materials such as mortars.

In Reference [22], Pavlik et al. tested mortars based on hydrated lime (HL), natural hydraulic lime (NHL) and a mixture of Portland cement and lime (PCHL). In the case of hardened mortar samples, the complete set of structural, mechanical and thermal properties was experimentally assessed. The moisture content had a significant influence on the thermal properties of porous materials [22,23]. This is due to the high thermal conductivity of water compared to air which fills the pores of the materials. If the water only fills part of the pores, the cavities contain moist air with different thermal properties than dry air. The researchers observed, inter alia, that the use of lava aggregate caused a significant decrease in the value of thermal conductivity compared to the control specimens. The newly developed mortars with lava granules, despite their high porosity, showed a slightly lower dependence of thermal conductivity on moisture content. The use of lava sand as aggregate significantly increased the open porosity of the tested mortars.

Mortars with the W/C ratio = 0.50 with the addition of tire rubber and processed waste porous glass were tested in Reference [24]. Porosimetric measurements, thermal conductivity and thermal diffusivity tests were carried out on the mortars using the ISOMET 2104 device. For the standard mortar composition, the result was 1.95 W/(mK). Mortars based on tire rubber showed lower thermal conductivity and diffusivity compared to the control specimens. Generally, an exponential decrease was observed for the conductivity and diffusivity with the decrease in specific gravity of the specimens.

Pereira et al. [25] presented an experimental study about the effects of exposure to elevated temperature on the properties of mortars produced with different insulating aggregates. The study focused on three types of thermal insulating aggregates: expanded clay, granulated expanded cork and silica aerogel. Mortars with the following W/C ratios were tested: 0.55, 0.65, 0.90 and 1.00. An ISOMET 2114 device was used to determine the thermal conductivity of the specimens. The test was performed using a surface probe placed on the surface of the specimen, which analyzes the thermal response to thermal impulses transmitted by the probe. It has been shown that in terms of thermal properties, after exposing the samples to the temperature of 250 °C, the thermal conductivity decreased. The authors explain this phenomenon with possible changes in the internal structure of the mortars, namely increasing porosity and microcracks. In Reference [25], the relationship between the density and thermal conductivity of mortars was described by an exponential function.

Lion et al. tested the mortars with a W/C ratio equal to 0.50 [26]. The aim of the work was to characterize the influence of temperature increase on hydraulic properties of cement-based materials. The researchers’ results show a clear increase in porosity for specimens processed at 150 and 250 °C. This proves the mortar’s sensitivity to temperature and deep modifications of its porous structure.

The physical properties and durability of cement mortars were researched in Reference [27]. In addition to the strength parameters and resistance to chloride ions, the mortars were tested for porosity using the mercury intrusion porosimetry, also undertaken in this paper. Mercury intrusion was also used by the researchers in Reference [28] to determine the effect of the cork size on the pore size distribution and the total porosity of mortars with cork. Guarded hot plate apparatus was used to determine the thermal conductivity. Mercury porosimetry was also used in studies [29] of the influence of nanosilica on, inter alia, the thermal conductivity and pore characteristics of lightweight concrete. A minimal decrease in the thermal conductivity of samples containing a higher amount of nanosilica was observed. Moreover, it was found that the inclusion of nanosilica contributed to a significant reduction in the pore diameters of the microstructure.

Analyzing the above-mentioned papers, undertaken research and the used measurement techniques, we decided to assess the thermal and structural parameters of cement mortars, differentiated by the type of cement, W/C ratio and the level of moisture content. Therefore, in this paper, we examined and compared the properties of mortars based on three kinds of cement, additionally differentiated by type of W/C ratio. The paper analyzes, among others, the influence of moisture of nine tested mortars on their thermal properties. The list of essential objectives of our study is presented below:

- How does the used cement affect the microstructural parameters of the mortars?
- What is the impact of W/C ratio on the thermal parameters of the tested mortars?
- How will the thermal properties change in different moisture content state of the mortars?
- What is the effect of W/C ratio and used cement on the water absorbability of each mortar?

## 2. Materials and Methods 

This study attempts to assess the relations between the thermal parameters of cement mortars based on various cements with a different water and cement ratio and their microstructure. Three types of cements were used to prepare the specimens, namely CEM I 42.5R, CEM II A-S 52.5N and CEM III A 42.5N. All types of cements were taken from the same manufacturer (Heidelberg Cement Group, Górażdże, Poland). The characteristics of each cement are shown in the Table 1. 

**Figure 1:**
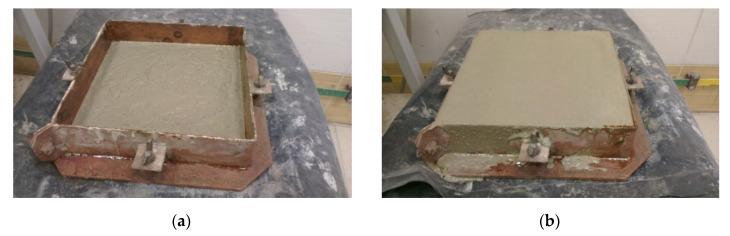
Filling the mold with mortar (**a**); Form with mortar after compaction (**b**).

From each of the cements that were used, three specimens with different water-to-cement ratios were made. These had the following values of W/C: 0.50, 0.55 and 0.60. The compositions of the mortars with W/C equal to 0.5 had a standard ratio of 3:1:0.5 (natural sand:cement:water). In the case of specimens with W/C ratios of 0.55 and 0.60, the sand to cement ratios were also constant and equal to 3 to 1. The mortars were based on natural aggregate with a grain diameter not exceeding 2 mm, which was dried at 70 °C. In total, nine specimens were prepared with dimensions of 25 × 25 × 6 cm^3^ (Figure 1). Large surface samples were prepared so that all thermal measurements could be made on one surface on each of the specimens.

Mortar-filled molds were stored for 24 h in containers on grates over water. The humidity level was over 90% and the temperature was 20 ± 1 °C. After demolding, the specimens were further conditioned under these conditions. After 28 days of curing, the specimens were subjected to preparatory work [30]. To eliminate unintentional measurement errors due to inaccuracy in surface finish, all materials have been aligned on professional grinding machines. The specimens’ surfaces should adhere exactly to the measuring probes of the device. Otherwise, air gaps are formed at the interface of these surfaces which affect the result of the thermal measurements.

**Figure 2:**
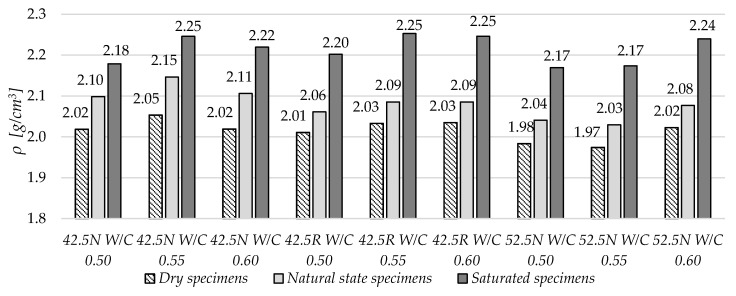
Volume density of dry, saturated and natural state specimens.

All the specimens’ dimensions were catalogued, and the samples were dried to constant mass at 70 °C in order to calculate the dry volume density of the materials. Then, the specimens were also saturated by direct immersion in water to determine their absorbability and density in the saturated state. The samples were then conditioned in air-dry conditions (humidity < 50% and temperature ~ 20 °C) for a period of 6 months. Then, their masses in the natural state were again determined. The results are summarized in Figure 2.

Based on the collected data, a small density variation in dry state can be observed for the nine mortars tested. The highest density in the dry state was obtained by mortar ZC 42.5N W/C 0.55, and the lowest by ZC 52.5N W/C 0.55. In turn, the highest density in the saturated state obtained the mortar ZC 42.5N W/C 0.55, and the lowest ZC 52.5N W/C 0.55. 

With the increase of the W/C ratio, the density of materials in the saturated state increased. For each of the tested cements, the proportions of these changes were different.

**Figure 3:**
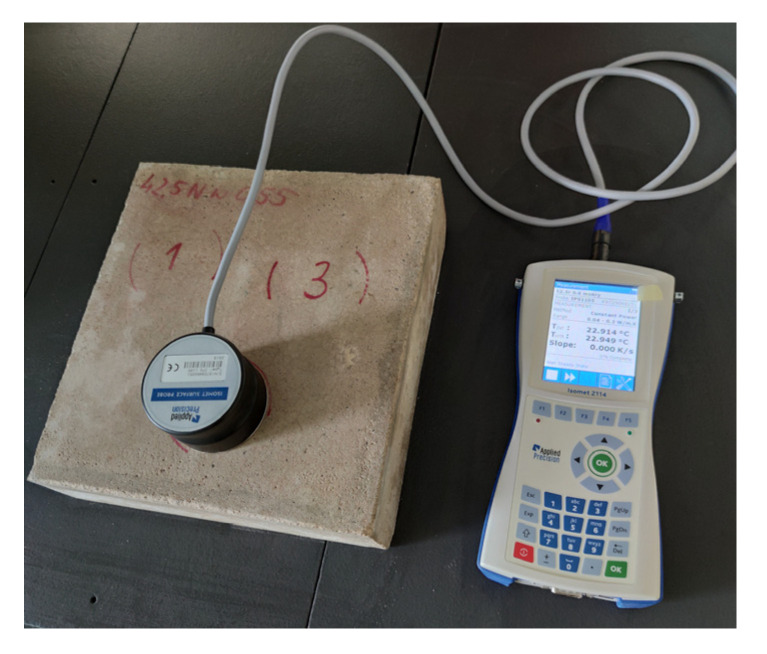
Thermal measurements test using the non-stationary heat flow method.

Tests of the changes of thermal parameters were performed using the nonstationary method. This technique is based on the analysis of heat flux readings at its non-stationary flow. The device uses surface probes or needle probes with a certain range of thermal conductivity. For the experiment, the surface probe (Applied Precision, Bratislava, Slovakia) of range from 0.3 to 2.0 W/mK was used. The probe heats the specimen with a specific heat flux and measures the thermal response in time. Thermal conductivity *λ*, volumetric specific heat *c_v_* and thermal diffusivity *a* were recorded. The tests were performed for the materials in dry, natural and saturated states. The testing was carried out on each specimen made from each type of mortar. The specimens were 6 × 25 × 25 cm in size, and the analyzed area was the bottom base of the specimen. Three measuring locations were marked on each of the six specimens to get the parameters from exactly the same place on the specimen each time. For each marked location, three measurements were conducted. For each specimen, nine measurements were done in total in each state. The mean values and standard deviations of the results were determined based on all the measurements. The prepared specimen is shown in Figure 3.

The specimens’ microstructure was analyzed using mercury intrusion porosimetry [31]. The mercury intrusion porosimetry (MIP) (Anton Paar, Graz, Austria) method can be successfully applied to assess the pore size distribution and fine pore structure in cement matrices, thus providing insight into their microstructural properties which are responsible for durability and moisture transport properties [29]. After 28 days of curing, cross-sections were cut from the specimens, which were then used to prepare 0.7 × 0.7 × 2.0 cm specimens. Glass cells of 2 cm^3^ volume were used. The specimens had approximately 1 cm^3^ volume, which is consistent with the standard ISO 15901-1. For each material, two specimens were analyzed. Before testing, the prepared specimens were dried in a laboratory dryer until constant weight was obtained. The surface tension of mercury was assumed to be 0.48 N/m and the contact angle was set at 140 degrees for the intrusion. Firstly, specimens were exposed to low pressure (up to 0.34 MPa), then mercury-filled cells with specimens were weighed. Secondly, the specimens were placed in a pressure chamber and subjected to high pressure (up to approximately 413 MPa). On the basis of obtained results of the injected mercury volume, the integral porosity graphs, log differential pore distributions, total porosity (in the research scope of the MIP method) and total specific surface area of specimens were determined.

## 3. Results 

**Figure 4:**
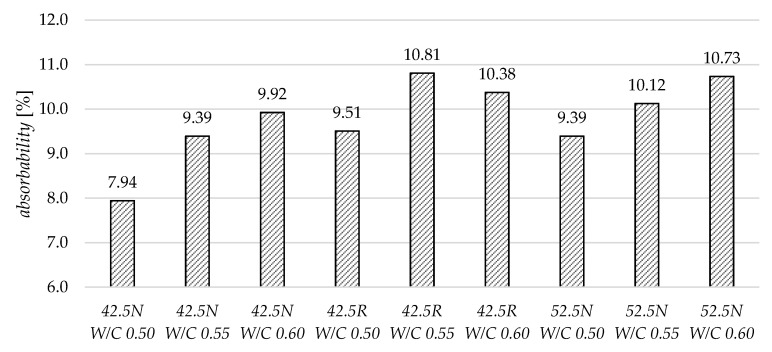
The comparison of specimens’ water absorbability.

The highest values of absorbability were achieved for mortar ZC 42.5R W/C 0.55, and the lowest for mortar ZC 42.5N W/C 0.5 (Figure 4). In two groups of mortars, ZC 42.5N and ZC 52.5N, a correlation was observed between the increase in absorbability and the increase in the W/C value. In the ZC 42.5R mortars, a small deviation from this dependence was observed for the mortar with the highest value of W/C = 0.6. There was a slight decrease in absorbability in this case in comparison to specimen W/C = 0.55.

**Figure 5:**
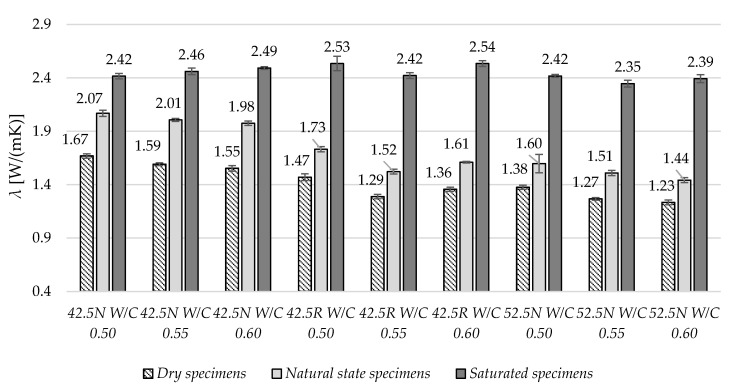
The average thermal conductivity values in three moisture states.

In Figure 5, the average values of thermal conductivity, *λ,* have been presented. In the case of dry and natural state specimens, with the increase of W/C ratio, the *λ* values decreased. In the variant of specimens fully saturated with water, no correlation was observed. In this variant, the *λ* values are in the range from 2.35 to 2.54 W/(mK). In the variant of dry specimens, the values are in the range from 1.23 to 1.67 W/(mK).

**Figure 6:**
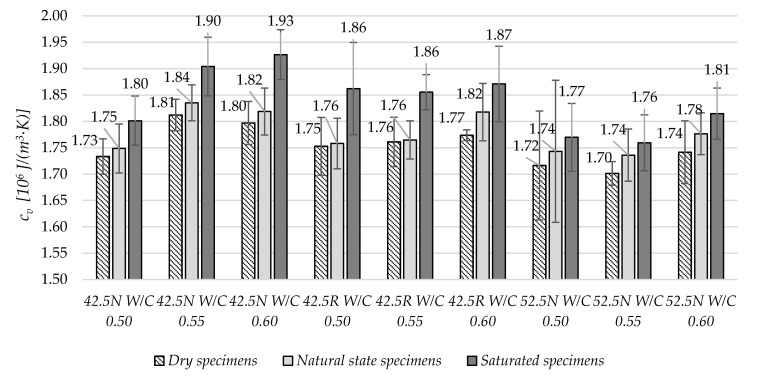
The average volumetric specific heat values in three moisture states.

Figure 6 shows the average values of volumetric specific heat. For dry state specimens, the average *c_v_* values were from 1.70 to 1.80 × 10^6^ J/(m^3^K). In case of fully saturated samples, the obtained values were in the range from 1.76 to 1.93 × 10^6^ J/(m^3^K). For the all specimen types in individual mortars, the increase in moisture level caused an increase in specific heat average values. For example, for the mortar CEM 42.5N W/C 0.5, the specific heat increased from dry to saturated state by nearly 4%. However, no direct correlation was found between the W/C of each of the nine groups of mortars and the change in the values of specific heat in different moisture states.

Generally, at higher values of the w/c ratio, an increase in volume specific heat was observed.

**Figure 7:**
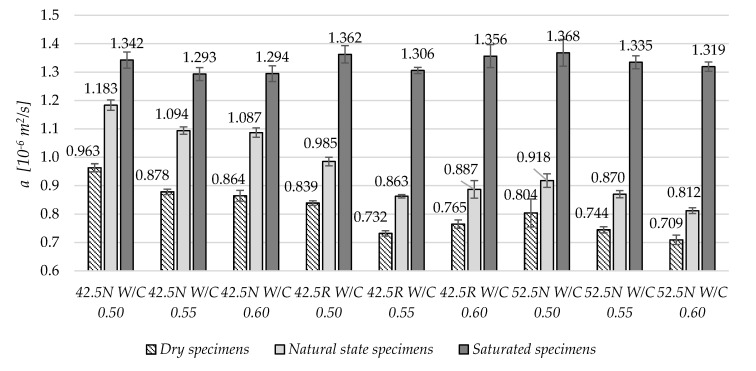
The average thermal diffusivity values in three moisture states.

The thermal diffusivity, *a,* similarly like thermal conductivity, *λ,* decreased with increased W/C ratio, as shown in Figure 7. This correlation is valid for specimens in dry and natural states. In case of specimens in saturated state, the differences between specimens are small and no correlation can be found. For dry materials, the range is from 0.71 to 0.96 × 10^−6^ m^2^/s, and for fully saturated specimens, the values are in the range from 1.29 to 1.37 × 10^−6^ m^2^/s.

**Figure 8:**
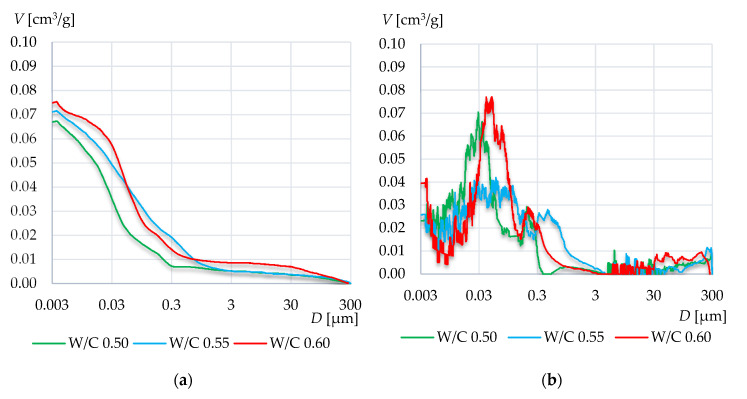
Cumulative distribution volume (**a**) and log-differential pore distribution (**b**) graphs of the mortars based on CEM III A 42.5N.

**Figure 9:**
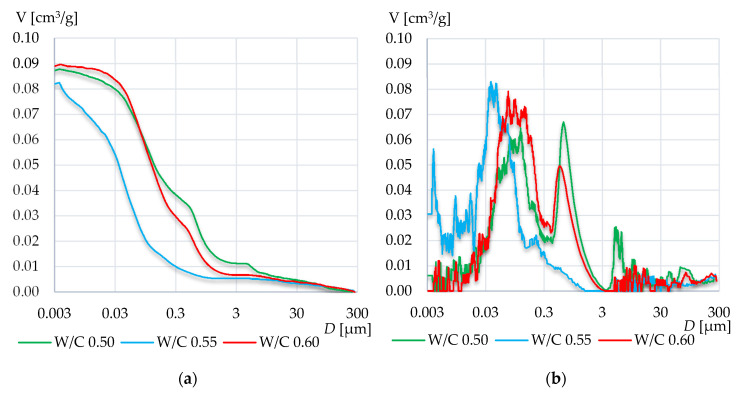
Cumulative distribution volume (**a**) and log-differential pore distribution (**b**) graphs of the mortars based on CEM I 42.5R.

**Figure 10:**
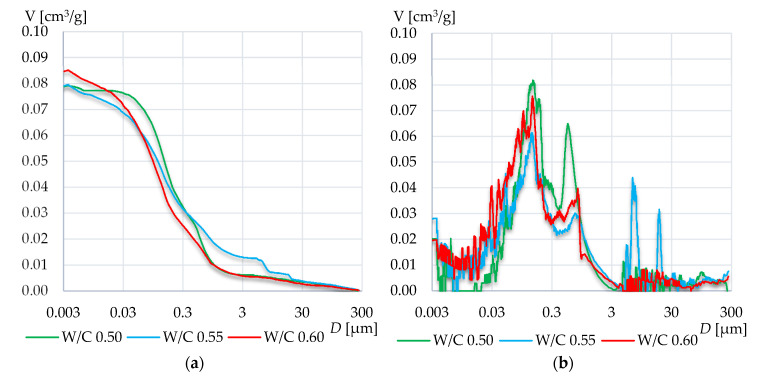
Cumulative distribution volume (**a**) and log-differential pore distribution (**b**) graphs of the mortars based on CEM II A-S 52.5N.

Figure 8, Figure 9 and Figure 10 present the cumulative distribution of pore volume and the log-differential pore distribution graphs for each of the tested specimens’ groups. In the first mortar group (CEM III A 42.5N), the increase of W/C ratio resulted in an increase of the total intruded mercury volume. In the case of mortar W/C 0.50, a high peak has occurred in the range from 0.02 to 0.06 μm. A similar peak has occurred in mortar W/C 0.60, however it is located from 0.03 to 0.10 μm. Presumably, the higher value of W/C resulted in diluting of the cement matrix and thus increased the number of pores smaller than 0.06 µm. As a result, there were fewer pores in the range from 0.06 to 0.06 µm, and a higher amount below 0.06 µm compared to the results of the mortar with W/C 0.55.

In case of the W/C 0.55, the pore log-differential graph was more evenly distributed and such high peak did not occur.

In case of mortars based on CEM I 42.5R, a much higher total intruded mercury volume has been observed than in CEM III A 42.5N. In this group, the graphs for W/C 0.55 are definitely different from the other two. In W/C 0.55, a high increase of pores of diameters less than 0.2 μm can be observed. The result is a significantly higher total specific surface area of the tested mortars of W/C 0.55 compared to other mortars. Moreover, the peak that appeared around 0.7 μm in W/C 0.50 and 0.60 has not occurred in this case. It has rather been shifted to the range from 0.03 to 0.3 and therefore, in this range, the highest porosity is visible compared to the other two mortars. Again, the higher cumulative intruded volume was recorded for the highest W/C ratio.

The differences among mortars based on cement CEM II A-S 52.5N are rather small. Again, with the increase in W/C ratio, the total intruded mercury volume increased. In all specimens, two peaks occurred, around 0.7 μm and 0.15 μm. Again, in the mortar with W/C 0.55, the peak value between 0.3 and 3 µm is the lowest. On the other hand, in this case, large pores with diameters ranging from 3 to 30 µm appeared.

**Figure 11:**
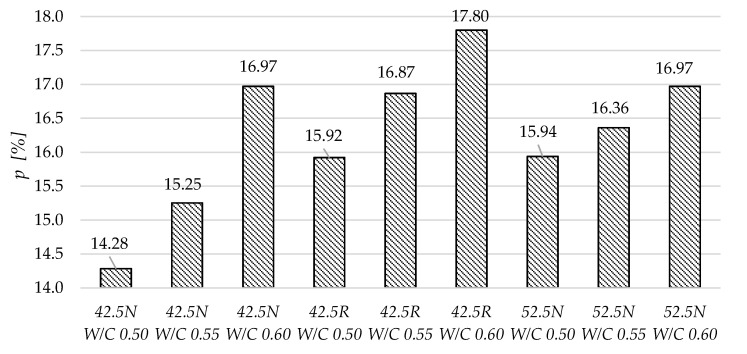
Total porosity of the tested mortars determined using mercury intrusion porosimetry.

**Figure 12:**
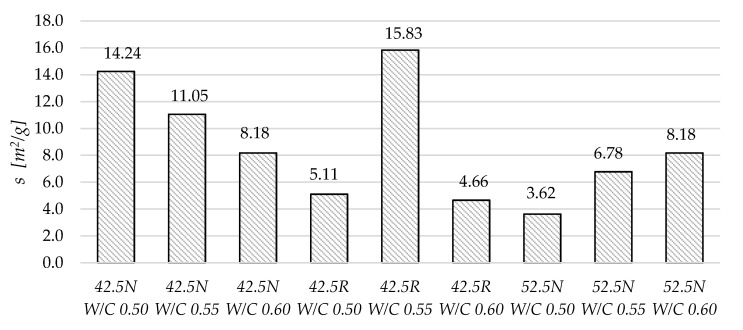
Total surface area of the tested mortars determined using mercury intrusion porosimetry.

Figure 11 and Figure 12 present the total porosity and total surface area values for the tested mortars. Generally, the highest porosity was observed in mortars with the highest W/C ratio. Some correlation between the W/C and total porosity can be observed. In case of the total surface area in each group, the relation between W/C and *s* is different. In mortars based on cement 42.5N, with the increase in W/C, the total surface area is decreasing. In mortars made of cement 52.5N, the increase in W/C resulted in an increase in surface area. In mortars based on CEM I 42.5R, the very high increase of total surface area can be observed in the specimen of W/C equal to 0.55, which is more than three times that in the other two specimens.

## 4. Discussion

**Figure 13:**
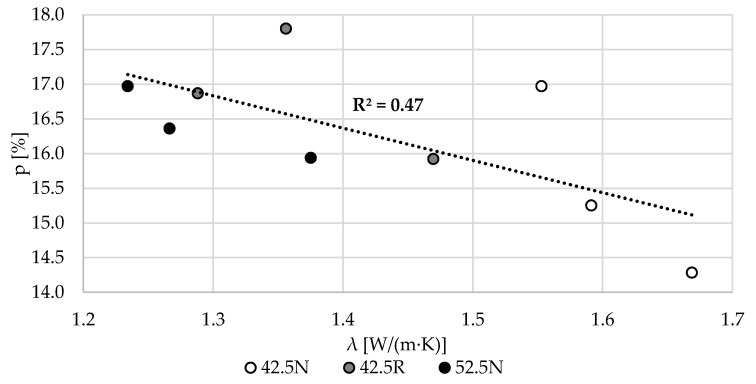
The relation between the thermal conductivity, *λ,* and total porosity, *p* (MIP).

In Figure 13, the relations between the thermal conductivity of the specimens and mercury intrusion porosimetry microstructural parameters are presented. In case of total porosity, the increase of porosity resulted in a reduction of thermal conductivity. The relation is strong for the variants based on cements 42.4N and 52.5N. In these variants, the coefficient of determination is higher than 0.8. In the group of specimens based on CEM 42.5R, the values for the specimen of W/C 0.55 varied substantially from the other results, thereby significantly reducing the coefficient of determination of the whole variant. In general, on the basis of data on all 9 mortars, the obvious dependence of thermal conductivity on porosity was confirmed.

The same situation occurred in the relation between the thermal conductivity and the total surface area. Again, in the variants based on CEM 42.5N and CEM 52.5N, the correlations are very strong. In the group 42.5N, with the increasing λ, the total surface area rises. In the group 52.5N, with the increasing λ, the total surface area decreases. In the 42.5R variant, the specimen W/C 0.55 is characterized by a definitely higher value of total surface area than the other two specimens. 

**Figure 14:**
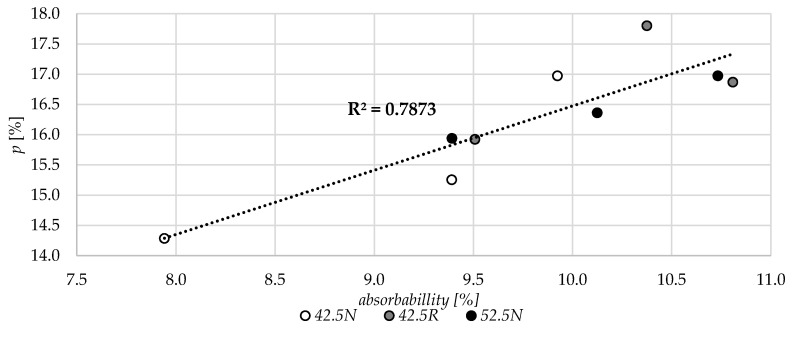
The relation between the water absorbability and total porosity, *p* (MIP).

In Figure 14, the relations between the water absorptivity and microstructural parameters are shown. With the increase of the porosity, the water absorbability increases for all three variants of the mortars. Overall, the coefficient of determination for all 9 specimens is quite high and confirms the relationship between the parameters.

In the variant made from cement 42.5N, the increase of total surface area resulted in a decrease of the specimen water absorbability. On the contrary, in variant of CEM 52.5N, the increase of total surface area caused the increase of water absorbability. A similar relation was observed in the CEM 42.5R group.

**Figure 15:**
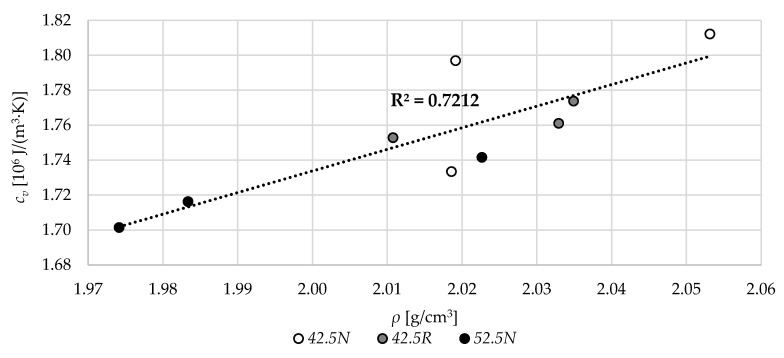
The relation between the dry volume density, *ρ,* and volume specific heat, *c_v_*.

In Figure 15, the relation between the dry volume density of specimens and their volume-specific heat in dry state is presented. Generally, the higher the volume density of a specimen, the higher the value of specific heat. The overall *R*^2^ for all 9 specimens was equal to 0.72.

**Figure 16:**
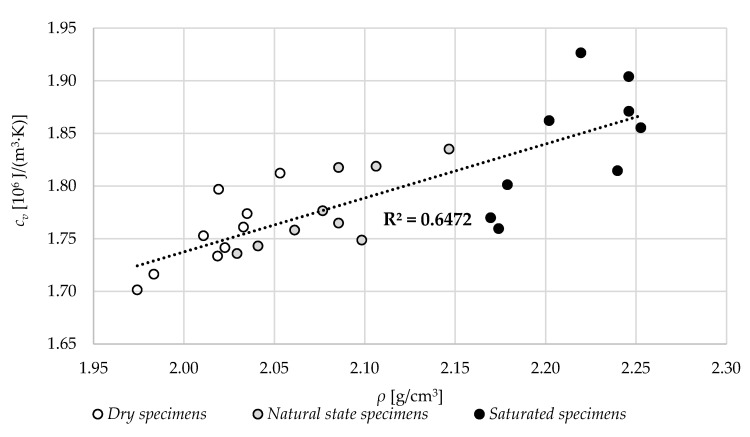
The relation between the volume density, *ρ,* in different specimen states and volume-specific heat, *c_v_*.

Similar results are presented in Figure 16; however, they apply to specimens with different moisture levels. Again, the higher the density of specimens, the higher the volume-specific heat. The increase in moisture content resulted in an increase of volume-specific heat of specimens. The *R*^2^ for all the data was 0.65.

**Figure 17:**
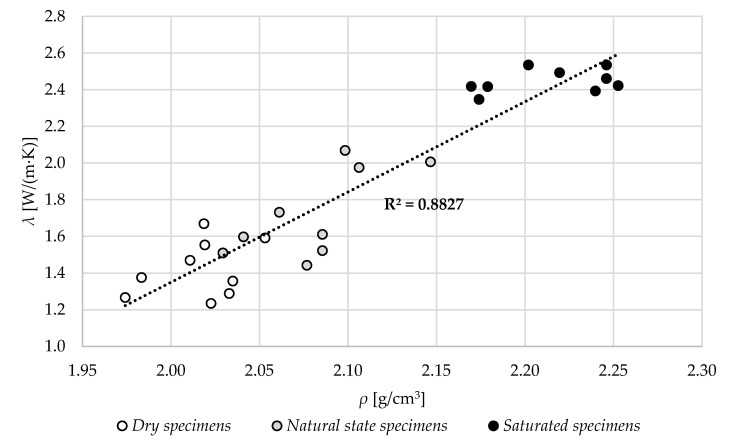
The relation between the volume density, *ρ,* in different specimen states and thermal conductivity, λ.

In Figure 17, the relation between the volume density and thermal conductivity for all the results is shown. With the increase in density, in all three specimens’ moisture states, the thermal conductivity increased. Overall, for the whole specimens in all states, the coefficient of determination is quite high and equal to 0.88.

**Figure 18:**
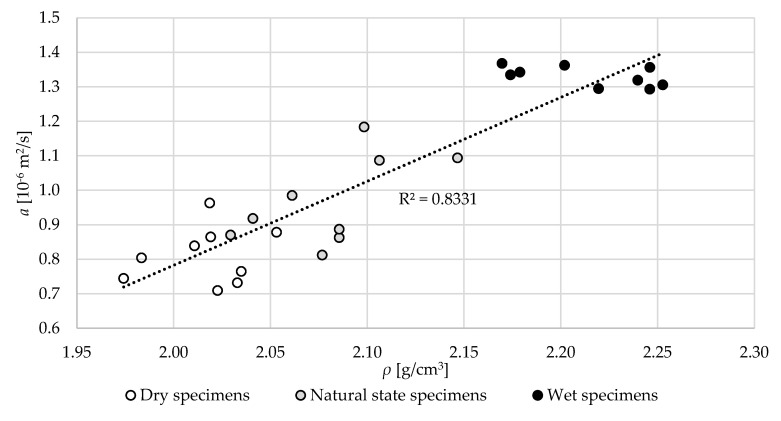
The relation between the volume density, *ρ,* in different specimen states and thermal diffusivity, *a*.

The relation between the volume density, *ρ,* in different specimen states and thermal diffusivity, *a,* is shown in Figure 18. Similar to thermal conductivity, the thermal diffusivity increases with the increase of specimen density. The moisture increase also resulted in an increase of thermal diffusivity.

**Figure 19:**
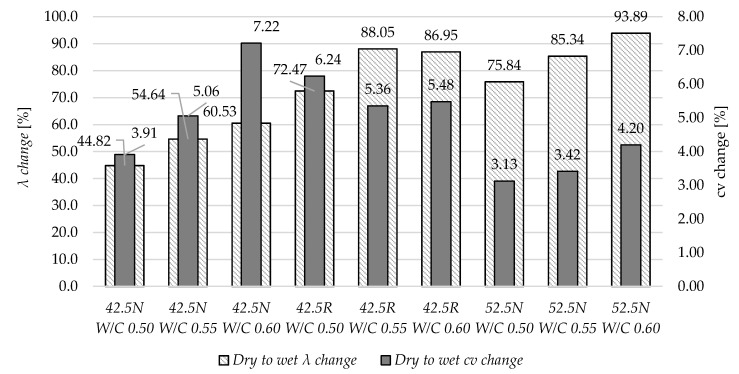
The relative change in the thermal parameter of specimens in dry and saturated state.

Figure 19 shows the relative change in thermal parameters between specimens in dry and water saturated states. In terms of thermal conductivity, the increase of W/C ratio generally resulted in the increase of relative parameter change. The exception is the specimen 42.5R W/C 0.55. This is probably due to the much higher specific surface area of this sample.

In variants made of cements 42.5N and 52.5N, the increase of W/C ratio also resulted in an increase of volume-specific heat *c_v_*. In the group based on CEM I 42.5R, the relation is inconclusive and the highest change was observed for specimen W/C 0.50.

## 5. Conclusions

This study examined the thermal parameters of mortars based on different cement type and W/C ratios. The results of the experimental studies can be summarized as follows:

- The cement used affects the relationship between the total specific surface area and the W/C ratio. In mortars based on CEM III A 42.5N, the total surface area decreased with the increased W/C ratio. The opposite relationship was observed in the mortars based on CEM II A-S 52.5N, where the surface area increased with the higher W/C ratio.
- As expected, the total porosity of specimens was higher for mortars with higher W/C ratios. The increase in porosity resulted in a reduction of thermal conductivity values and an increase of water absorbability of all tested mortar groups.
- Strong correlations between the thermal parameters and materials’ density were observed. The higher the values of volume density, the higher values of thermal conductivity and volume-specific heat were obtained. With the increase of specimens’ moisture content, the thermal parameters increased, as expected. In mortars based on CEM III A 42.5N and CEM II A-S 52.5N, the increase was proportional to W/C ratio.

Most building standards use fixed thermal properties for normalized building conditions. It is also often assumed that an increase in W/C ratio will clearly cause an increase in porosity and surface area of mortars and at the same time, a decrease of thermal properties. It has been proven in the publication that these relationships are much more complex and depend mainly on the cement used.

Therefore, the results presented in this article may demonstrate to designers and specialists the importance of mortar composition on its thermal properties. It also highlights the influence of moisture-related parameters and their effect on the problem of moistening the building components.

The obtained relationships can also be used in hygrothermal simulations of building components made on the basis of cement mortars of the tested compositions. This would increase the accuracy of the analyses and would allow to simulate the partitions in the real state of moisture content.

## Figures and Tables

**Table 1:** Characteristics of cements.

Cement Name	Composition	Surface Area (cm^2^/g)	Initial Setting Time (min)	SO_3_ (%)	Cl (%)	Compression Strength after 2 Days (MPa)	Compression Strength after 28 Days (MPa)
CEM I 42.5R	clinker content of 95% to 100%	3632	239	2.97	0.060	28.3	59.6
CEM II A-S 52.5N	clinker content of 80% to 94%; blast furnace slag content of 6% to 20%	4289	204	2.95	0.055	26.4	58.2
CEM III A 42.5N	clinker content from 35% to 50%; blast furnace slag content from 50% to 65%	4499	215	2.11	0.080	15.7	56.9
